# Optimizing Operating Parameters of High-Temperature Steam for Disinfecting Total Nematodes and Bacteria in Soil: Application of the Box–Behnken Design

**DOI:** 10.3390/ijerph17145029

**Published:** 2020-07-13

**Authors:** Da-An Huh, Woo Ri Chae, Hong Lyuer Lim, Joung Ho Kim, Yoo Sin Kim, Young-Whan Kim, Kyong Whan Moon

**Affiliations:** 1 Department of Health Science, Korea University, Anam-ro 145, Seongbuk-gu, Seoul 02841, Korea; black1388@korea.ac.kr; 2 Department of Health and Safety Convergence Science, Korea University, Anam-ro 145, Seongbuk-gu, Seoul 02841, Korea; wr245@naver.com (W.R.C.); limhl1213@naver.com (H.L.L.); 3 Department of Technology Research and Development, JSE Inc., Muyeol-ro 39, Suseong-gu, Daegu 42033, Korea; marujune7273@naver.com (J.H.K.); jse2013@naver.com (Y.S.K.); kywh@korea.ac.kr (Y.-W.K.)

**Keywords:** Box–Behnken design, high-temperature steam disinfection, total nematode, removal efficiency, total bacteria

## Abstract

Concerns about the widespread use of pesticides have been growing due to the adverse effects of chemicals on the environment and human health. It has prompted worldwide research into the development of a replacement to chemical disinfection of soil. The efficiency of steam sterilization, an alternative to chemical methods, has improved as technology has advanced, and the Agricultural Research and Extension Service in Korea recommends the use of steam sterilization. However, few studies have been conducted on the effects and operating conditions of high-temperature steam disinfection. In this study, we present the optimum operating conditions of a high-steam disinfector, to maximize the cost-effectiveness and removal efficiency of total nematodes and total bacteria in soil using the Box–Behnken design. The experimental data were fitted to a second-order polynomial equation using multiple regression analysis, with coefficients of determination ($R^{2}$) for each model of 0.9279, 0.9678, and 0.9979. The optimum conditions were found to be a steam temperature of 150.56 °C, running speed of 1.69 m/min, and spray depth of 15.0 cm, with a corresponding desirability value of 0.8367. In the model, these conditions cause the prediction of the following responses: nematode removal efficiency of 93.99%, bacteria removal efficiency of 97.49%, and oil consumption of 70.49 mL/m2. At the optimum conditions for the steam disinfector, the removal efficiencies of nematodes and bacteria were maximized, and the oil consumption was minimized. The results of our study can be used as basic data for efficient soil disinfection using high-temperature steam.

## 1. Introduction

Plant-parasitic nematodes and plant-pathogenic bacteria in soil can survive in the soil for a long time and cause serious damage to crops [1,2]. In Korea, approximately 42% of the crop plantations that were surveyed during 2013–2015 were infected with *Meloidogyne* [3], which absorbs nutrients from the crops and creates galls in the roots causing malnutrition in the crops [4]. The number of insect pests and bacteria has been increasing because of global warming [5]. Farming households have been disinfecting soil to prevent crop damage using chemicals, and soil fumigation is commonly performed. However, concerns about the widespread use of pesticides have increased as the adverse effects of chemicals on the environment and human health have become known. Methyl bromide, a representative soil fumigant, has been prohibited across the world since 2015 because it has highly toxic and ozone-depleting properties [6]. Although relatively less harmful pesticides such as pyrethroid pesticides have been used, the health effects of these substitutes were reported recently [7]. As a result, this has prompted worldwide research into the development of a replacement for chemical disinfection of soil [6,8]. 

Steam sterilization, presented as one of the alternatives to chemical methods, is the oldest physical method and has been applied for more than a century [9]. Steam treatment needs a large machine that can generate steam and introduce it into soils, and the efficiency of this method depends upon the soil type because soil temperatures are only increased in the upper soil layers in the case of sandy and loam soils [10]. Despite these obstacles, the efficiency of soil sterilization using high-temperature steam is gradually improving. Recent technological improvements have made steam sterilizers lighter and faster [11]. In addition, the steam generator has become more energy efficient as the technology to reliably spray steam deeper into the soil has been developed [12]. Above all, the main advantages of steaming are the relative simplicity of the method and the high sterilization efficiency in the case of most plant pathogens, insects, viruses, and weed seeds in the soil [9,10]. 

In 2011, chemical disinfectants used with household humidifiers caused unidentified fatal lung diseases in Korea. According to the Ministry of Environment, this chemical misuse caused about 7000 victims by 2020. The incident prompted people to reduce the use of chemicals not only in their daily lives but also in agriculture [13]. In addition, the Agricultural Research and Extension Service in Korea recommended eco-friendly steam sterilization and has developed a high-temperature steam disinfector for use by farmers. However, some farming households are overusing steam under unoptimized conditions, which can increase the cost of soil disinfection over chemical methods. Few studies have been conducted on the manipulation of the operating conditions to increase the disinfection efficiency [12,14]. To perform efficient soil disinfection, it is necessary to optimize the operating conditions to increase the disinfection efficiency and cost-effectiveness.

The aim of this study is to present the optimum operating conditions of high-temperature steam that maximize the disinfection efficiency and the cost-effectiveness for the disinfection of total nematodes and total bacteria in soil. In this study, we present the optimal operating conditions for high-temperature steam sterilization using the Box–Behnken design and the removal efficiency and cost-effectiveness for nematodes and bacteria in soil under the optimized conditions. The results of our study can be used as basic data for efficient soil disinfection using high-temperature steam.

## 2. Materials and Methods 

### 2.1. Soil Sterilization and Sampling

We used a high-temperature steam disinfector (JS-S002A, JSE Inc., Daegu, Korea) to conduct soil disinfection. The size of the device is 2510 mm (length) × 1600 mm (width) × 1270 mm (height), and it can disinfect soil at 100 m^2^ per hour. This disinfector can spray high-temperature steam into the soil by using needles with spraying nozzles, and reliably spray steam at 120–160 °C degrees. An advantage of this device is that it consumes less energy than others because the hot steam sprayed into the soil rises after combining with geothermal heat. Soil disinfection was carried out between 10 a.m. and 12 a.m. in a greenhouse during the nongrowing season.

For recent decades in Korea, nematodes and bacteria in the soil have been pointed out as the main reasons for disrupting crop production [5]. Therefore, in this paper, the total nematodes and total bacteria were chosen as indicator pests. To analyze the density reductions of total nematode and total bacteria caused by disinfection, we collected soil before and after running the steam disinfector. We conducted experiments in the greenhouse to minimize the effects of temperature and humidity. A set of 15 greenhouses in Miryang, Gyeongsang Province, Korea, were selected as test sites. The main crop cultivated in the greenhouses was red peppers, and at the time of the experiment, these greenhouses were in an uncultivated period. No disease history was reported caused by nematode or bacteria in these 15 greenhouses, and the ranges of baseline populations of total nematode and total bacteria before steam treatment across the 15 greenhouses were 135–233 nematodes/30 g soil and 2.27 × 10^11^–5.32 × 10^12^ CFU/g soil, respectively. All greenhouses were the same size at 200 m^2^, and it took about four hours to treat each greenhouse using a steam disinfector. We randomly selected seven points in each greenhouse before and after steaming and evenly collected 200 g of soil with a depth of 0–15 cm at each point. The collected soils were not completely sealed for airflow, and we maintained the existing soil temperature using iceboxes. All soil was transferred to the laboratory within six hours after sampling.

### 2.2. Calculation of the Removal Efficiency of Total Nematodes and Total Bacteria

We analyzed the effects of high-temperature steam disinfection by comparing the density of total nematodes and total bacteria before and after soil disinfection. For the analysis of the density of nematodes, the commonly used Baermann funnel method was used to isolate living nematodes [15]. Three replicate experiments were conducted with 30 g of mixed soil from the seven collection points. We counted nematodes gathered under the funnel after passing through facial tissue, using an optical microscope (Olympus BX41TF, Olympus Optical Co., Tokyo, Japan). The analysis of the density of total bacteria in the soil was performed using the dilution plate method after extracting 100 g of the mixed soil [16]. We conducted three repeated experiments with dilution factors of 10^−6^–10^−10^ using TSA culture. The diluted Petri dishes were incubated for 48 h at 25 °C, and the colonies on the Petri dishes were counted.

After counting the number of nematodes and bacteria, before and after disinfection, we calculated the removal efficiency according to the following equation:

(1) $$\mathrm{RE}\;\left(\%\right)=\frac{N_{B}-N_{A}}{N_{B}}\times 100$$

where RE is the removal efficiency, $N_{B}$ is the number of nematodes or colonies before the steam sterilization, and $N_{A}$ is the number of nematodes or colonies after the steam disinfection.

### 2.3. Measurement of Energy Consumption

We calculated the amount of light oil consumed during soil disinfection to estimate the energy consumption of high-temperature steam disinfection. The oil consumption was estimated by calculating the difference between the amount of light oil before and after disinfection. We divided this amount by the area of the disinfected field and calculated the amount of light oil consumed per unit area of the greenhouse.

### 2.4. Selection of Target Parameters for Optimization

When using a hot steam soil disinfector in farming households, the parameters that can be easily controlled are the temperature of the steam, the speed of the disinfector, and the depth of steam injection. These variables are related to the removal efficiency of nematodes and bacteria, and also to the cost of disinfection. Therefore, we selected these three variables as optimization target variables in this study. In addition, since the high-temperature steam disinfector may not be able to control the steam spraying depth due to the condition of the soil, we also investigated the optimum ranges for the steam temperature and running speed of the disinfector at each of the steam spraying depths.

### 2.5. Response Surface Methodology and Box–Behnken Design

Response surface methodology (RSM) is a collection of statistical and mathematical techniques for designing experiments, constructing models, and evaluating the effects of process parameters. The RSM uses a series of designed experiments to obtain the optimal response, which is then modeled using a quadratic polynomial. The response surface can be graphically utilized to determine the relationship between the explanatory and response variables [17], and this visual representation allows the user to better understand the properties of the relationships between the variables [18]. Knowledge of this relationship is important for finding the conditions of the variables that provide the optimal response [19]. 

The Box–Behnken design (BBD), one of the RSM designs, is an independent and rotatable quadratic design with variable combinations at the midpoints of the edges and the center of the experimental space [20]. The BBD requires three levels for each explanatory variable, which results in fewer experimental trials being required, compared to the other RSM designs, to estimate the effects of variables and their interactions [21]. Therefore, we designed experiments to optimize the variables using the BBD.

We established experiments based on the BBD with three factors at three levels. For statistical calculation, each explanatory variable was coded at three levels: −1, 0, and 1. The coding of the variables was conducted using the following equation:

(2) $$X_{i}=\frac{x_{i}-x_{c}}{\Delta x_{i}}\;\;\;\;\;\;\;\;i=1,\;2,\;3,$$

where $X_{i}$ is the dimensionless coded value of an independent variable, $x_{i}$ is the actual value of an independent variable, $x_{c}$ is the actual value of an independent variable at the center point, and $\Delta x_{i}$ is the step change value of an independent variable.

In the BBD, a total number of 15 experiments, including three center points, were conducted. A second-order polynomial model was fitted to correlate the relationship between the response and explanatory variables. We estimated the goodness-of-fit and coefficients of each model using the statistical software Minitab (version 19, Minitab Inc., State College, PA, USA). A quadratic model that includes the linear model is:

(3) $$Y=\beta_{0}+\overset{3}{\underset{i=1}{\sum }}\beta_{i}X_{i}+\overset{3}{\underset{i=1}{\sum }}\beta_{ii}X_{i}^{2}+\overset{2}{\underset{i=1}{\sum }}\overset{3}{\underset{j=2}{\sum }}\beta_{ij}X_{i}X_{j}+e_{i}$$

where Y is the response variable, $X_{i}$ and $X_{j}$ are the explanatory variables, $\beta_{0}$ is the model constant, $\beta_{i}$ represents the linear coefficient, $\beta_{ii}$ denotes the quadratic coefficient, $\beta_{ij}$ indicates the interaction coefficient, and $e_{i}$ is the statistical error.

### 2.6. Steps for Parameter Optimization

The following steps were used to optimize the operation parameters of the steam soil disinfector (Figure S1; a flow chart diagram). For the process of optimization, the steam temperature, running speed, and spraying depth were used as the explanatory variables, and the removal efficiency of nematodes and total bacteria, as well as the consumption of light oil were used as the response variables.

Step 1. Observe the temperature changes of the soil according to the level of the three explanatory variables. In the BBD, the range of each variable should include the changing point at which the result begins to increase or decrease. Therefore, if the range of each variable does not include a changing point at which the degree of increase in the soil temperature begins to decrease, reset the range of the variables. If the range includes the changing point, set three levels of these variables for performing the BBD. These pre-experiments were performed sequentially by dividing the same greenhouse of 400 m^2^ into four parts. Temperatures of 9 cm, 12 cm, and 15 cm depth under the soil were measured simultaneously at five-minute intervals using digital thermometers (model CS-101, ACUBA, Seoul, Korea).
Step 2. Conduct the BBD experiments and calculate the removal efficiency of nematodes and total bacteria, and the amount of light oil used for soil disinfection.
Step 3. Conduct the RSM simulation and analysis of variance (ANOVA) and estimate quadratic equations for the variables.
Step 4. Investigate the optimal conditions for the explanatory variables based on the contour graph and surface plots produced from the RSM simulations. The desirability values which represent the closeness of a response to its ideal value are calculated using the desirability function provided by Minitab software [22].

## 3. Results

### 3.1. Setting Ranges for the Explanatory Variables through Basic Experiments

**Figure 1:**
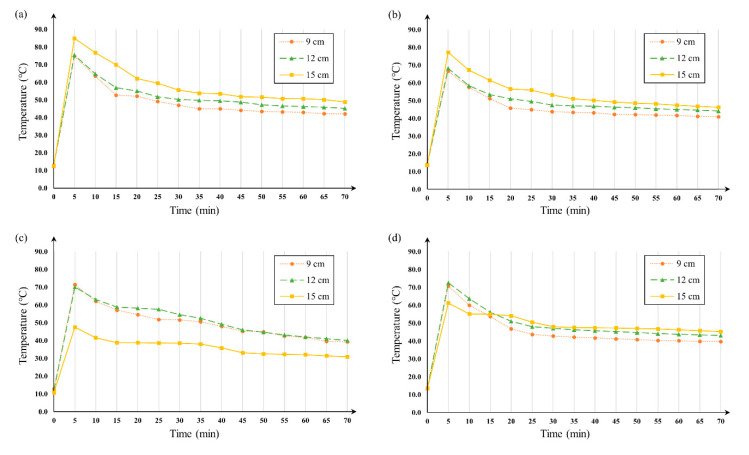
Temperature changes over time after steam disinfection: (**a**) 160 °C, 1 m/min, and 15 cm, (**b**) 120 °C, 1 m/min, and 15 cm, (**c**) 160 °C, 2 m/min, and 15 cm, and (**d**) 160 °C, 1 m/min, and 9 cm.

To set the ranges of the explanatory variables for the BBD, we measured the soil temperature by depth after steam disinfection with different variable levels. Figure 1 shows the changes in the soil temperature over time after steam disinfection. When the soil disinfector was operated at a steam temperature of 160 °C, a running speed of 1 m/min, and a spray depth of 15 cm, the soil temperature rose to 71.9 °C, 73.5 °C, and 83.5 °C at 9 cm, 12 cm, and 15 cm, respectively, and then gradually decreased (Figure 1a). The degree of decrease in the soil temperature decreased with time, and the soil temperature converged at 40 °C. In the subsequent experiments with different variable levels, the changing trends in the soil temperature at 9 cm and 12 cm were almost the same, but differences were found at 15 cm. At 15 cm, the degree of increase in the initial soil temperature decreased by about 5 °C when the steam temperature was lowered to 120 °C (Figure 1b) and reduced to less than half when the running speed was increased to 2 m/min (Figure 1c). We also found that the soil temperature at 15 cm only reached up to 60 °C when spraying steam at a depth of 9 cm. Based on these results, we set the three levels of explanatory variables in the BBD, as shown in Table 1.

### 3.2. BBD Experiments and Regression Models

Table 2 shows the experimental design according to the BBD and the results of the response variables in each experiment. The three-factor and three-level BBD required a total of 15 experimental runs. We performed a series of 15 experiments based on the experimental runs, designed with different combinations of the variable levels. Each greenhouse was used for each run. The ranges of nematode removal efficiency, total bacteria removal efficiency, and consumption of light oil were 82.56–97.25%, 80.23–96.83%, and 31–191 mL/m^2^, respectively. The removal efficiency of nematodes and total bacteria tended to increase as the oil consumption per unit area increased.

Table 3 shows the results of the ANOVA, and the goodness-of-fit of the models are also presented. In the ANOVA, the p-values for the models of the three response variables are 0.022, 0.003, and less than 0.001, respectively, and all models had a nonsignificant lack-of-fit. Therefore, it can be concluded that all response variables were fitted well by the quadratic model. The coefficients of determination ($R^{2}$), which indicate how much the model can predict variabilities in the response variables, are 0.9279, 0.9678, and 0.9979, respectively.

Empirical relationships between the three explanatory variables and responses were expressed by second-order polynomial equations with interaction terms. The final equations obtained in uncoded factors are as follow:

(4) $$\begin{array}{ll} RE_{nematode}\;\left(\%\right)= & 90.0+0.350x_{1}-30.2x_{2}-0.09x_{3}-0.00199x_{1}^{2}-4.12x_{2}^{2}\\ & -0.069x_{3}^{2}+0.1510x_{1}x_{2}+0.0045x_{1}x_{3}+1.085x_{2}x_{3} \end{array}$$

(5) $$\begin{array}{ll} RE_{bacteria}\;\left(\%\right)= & -1.2+1.883x_{1}-27.5x_{2}-2.75x_{3}-0.00786x_{1}^{2}-10.97x_{2}^{2}\\ & +0.0048x_{3}^{2}+0.1962x_{1}x_{2}+0.0059x_{1}x_{3}+1.888x_{2}x_{3} \end{array}$$

(6) $$\begin{array}{ll} OC\;\left(mL/m^{2}\right)= & -469+8.04x_{1}-83.8x_{2}+5.31x_{3}-0.01240x_{1}^{2}+84.17x_{2}^{2}+0.144x_{3}^{2}\\ & -1.925x_{1}x_{2}-0.0417x_{1}x_{3}-0.67x_{2}x_{3} \end{array}$$

where RE is the removal efficiency, OC is the oil consumption, $x_{1}$ is the steam temperature, $x_{2}$ is the running speed, and $x_{3}$ is the spray depth.

### 3.3. Response Surface and Contour Plot Analysis

**Figure 2:**
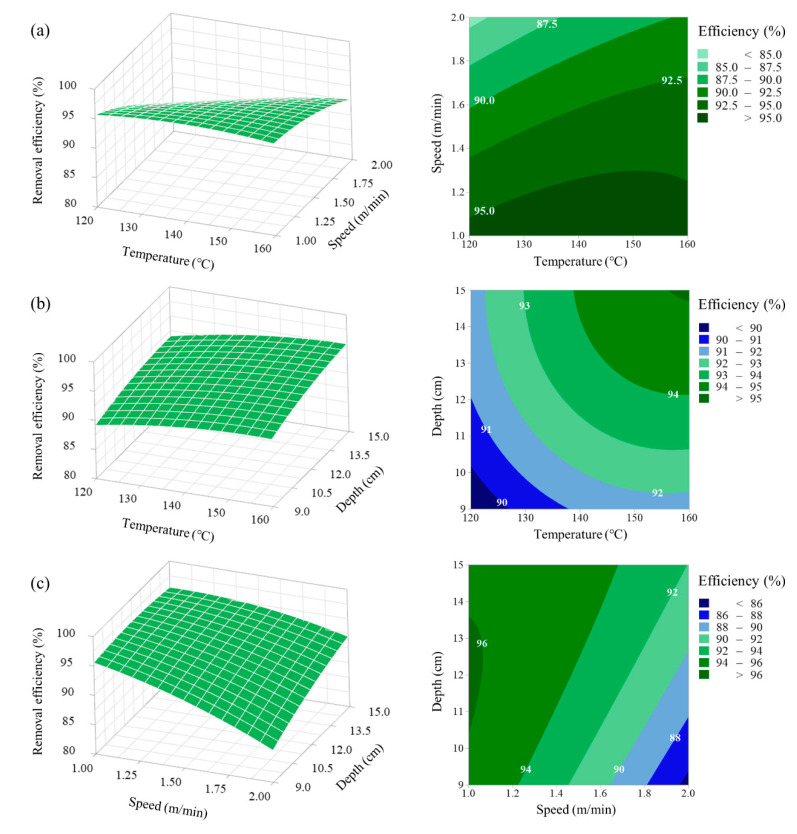
Response surface plots presenting the interaction effects of three variables on the total nematode removal efficiency: (**a**) surface plot showing the effect of the steam temperature and running speed on the removal efficiency, (**b**) surface plot showing the effect of the steam temperature and spray depth on the removal efficiency, and (**c**) surface plot showing the effect of the running speed and spray depth on the removal efficiency.

**Figure 3:**
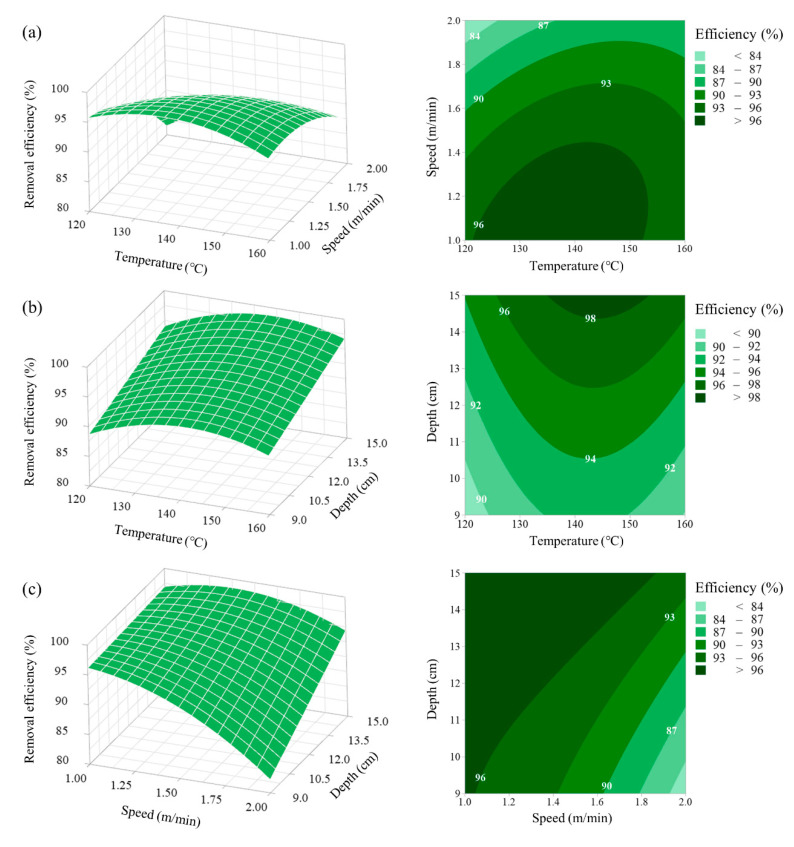
Response surface plots presenting the interaction effects of three variables on the total bacteria removal efficiency: (**a**) surface plot showing the effect of the steam temperature and running speed on removal efficiency, (**b**) surface plot showing the effect of the steam temperature and spray depth on removal efficiency, and (**c**) surface plot showing the effect of the running speed and spray depth on removal efficiency.

**Figure 4:**
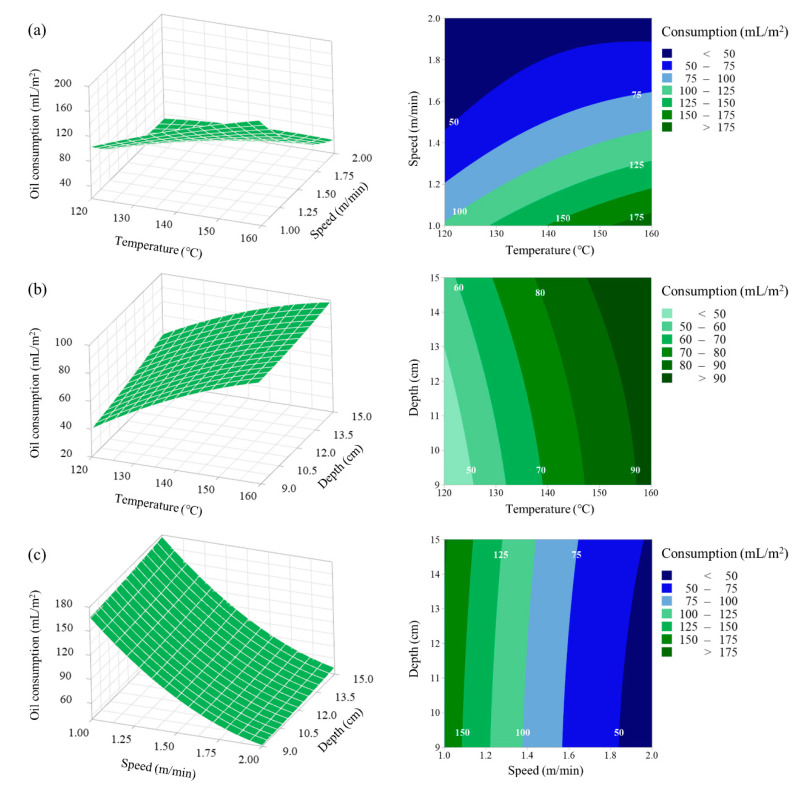
Response surface plots presenting the interaction effects of three variables on oil consumption: (**a**) surface plot showing the effect of the steam temperature and running speed on oil consumption, (**b**) surface plot showing the effect of the steam temperature and spray depth on oil consumption, and (**c**) surface plot showing the effect of the running speed and spray depth on oil consumption.

Figure 2, Figure 3 and Figure 4 present the three-dimensional response surface plots and two-dimensional contour plots, which explain the relationship between the explanatory and response variables. These plots can visualize the interaction effects between variables and provide information on how one factor is influenced by the change of another. Figure 2 shows the effects of two explanatory variables and their interactions with the response of nematode removal efficiency at the middle level of another variable. At the high steam temperature level (160 °C), the removal efficiency decreased from 95.54 to 90.16% when the running speed was increased from 1.0 to 2.0 m/min. At the low steam temperature level (120 °C), the removal efficiency decreased from 95.63 to 84.21% when the running speed was increased from 1.0 to 2.0 m/min (Figure 2a). The other interaction was observed between running speed and spray depth. At the high running speed level (2 m/min), the removal efficiency increased from 84.20 to 90.52% when the spray depth was increased from 9 to 15 cm. However, at the low running speed level (1 m/min), few changes were observed in the removal efficiency (Figure 2c).

Figure 3 shows the effects of two explanatory variables and their interactions with the response of total bacteria removal efficiency at the middle level of another variable. Similar to the response of the nematodes, interactions between the steam temperature and running speed and between the running speed and spray depth were observed. At the high steam temperature level (160 °C), the removal efficiency decreased from 93.46 to 87.10% when the running speed was increased from 1.0 to 2.0 m/min. At the low steam temperature level (120 °C), the removal efficiency decreased from 95.49 to 81.28% when the running speed was increased from 1.0 to 2.0 m/min (Figure 3a). In addition, at the high running speed level (2 m/min), the removal efficiency increased from 81.48 to 93.28% when the spray depth was increased from 9 to 15 cm. However, at the low running speed level (1 m/min), few changes were observed in the removal efficiency (Figure 3c).

Figure 4 presents the effects of two explanatory variables and their interactions with the response of oil consumption at the middle level of another variable. In the case of oil consumption, only one interaction between steam temperature and running speed was observed. At the high steam temperature level (160 °C), the oil consumption decreased from 188.68 to 41.35 mL/m^2^ when the running speed was increased from 1.0 to 2.0 m/min. At the low steam temperature level (120 °C), the oil consumption decreased from 102.98 to 32.65 mL/m^2^ when the running speed was increased from 1.0 to 2.0 m/min (Figure 4a).

### 3.4. Optimization of the Sterilization Operation Parameters

After studying the effects of the explanatory variables on the response variables, we simultaneously optimized the three explanatory variables using the desirability function. We aimed to maximize the nematode and bacteria removal efficiency and minimize the oil consumption. Equal weight was given to all response variables, and the overall desirability value was calculated by combining the individual desirability functions using the Minitab software. Table 4 presents the final settings for the optimal operation parameters. The optimized equation was achieved at a steam temperature of 150.56 °C, running speed of 1.69 m/min, and spray depth of 15.0 cm, with a corresponding desirability value of 0.8367. These conditions predicted the responses as follows: nematode removal efficiency of 93.99%, total bacteria removal efficiency of 97.49%, and oil consumption of 70.49 mL/m^2^.

**Figure 5:**
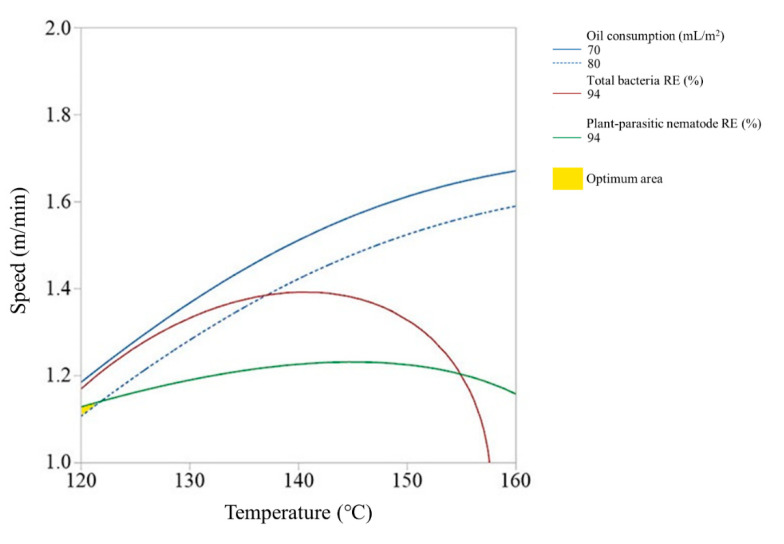
Overlay plot of the steam temperature and running speed on the response variables when the spray depth is 9 cm.

**Figure 6:**
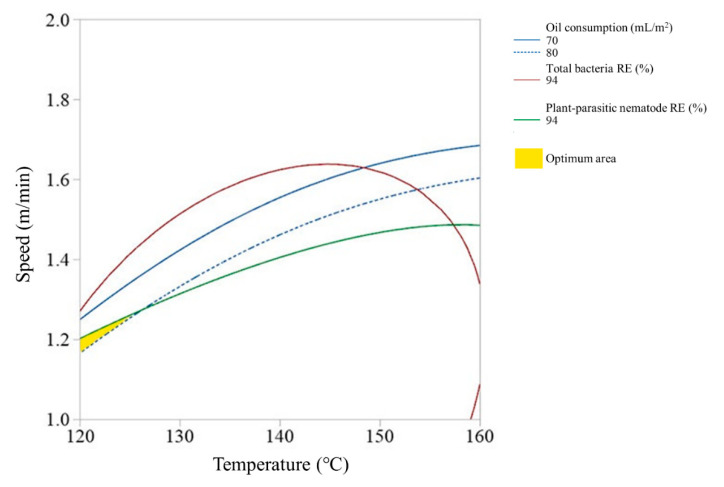
Overlay plot of the steam temperature and running speed on the response variables when the spray depth is 12 cm.

**Figure 7:**
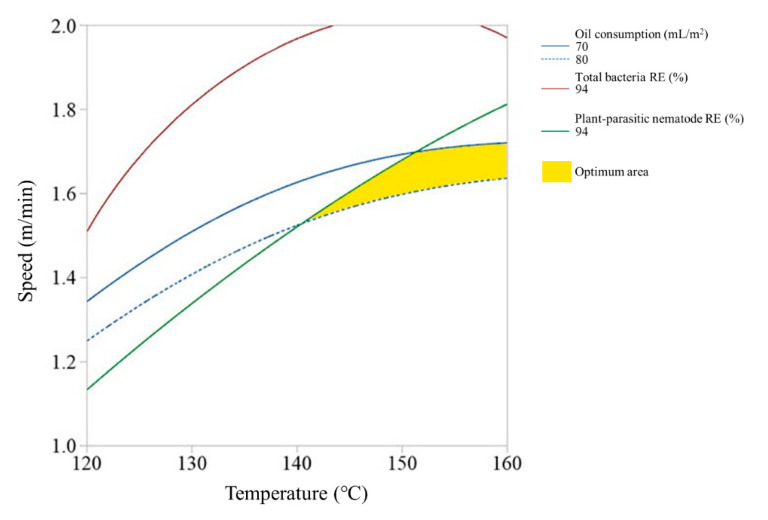
Overlay plot of the steam temperature and running speed on the response variables when the spray depth is 15 cm.

Since the high-temperature steam sterilizer may not be able to control the steam spraying depth due to the soil condition, we also investigated the optimum ranges of steam temperature and running speed of the disinfector at each of the steam spraying depths. Figure 5, Figure 6 and Figure 7 show the overlay plots of the effects of the steam temperature and running speed on the response variables at each spray depth. We set the target ranges of nematode removal efficiency, total bacteria removal efficiency, and oil consumption to 94–100%, 94–100%, and 70–80 mL/m^2^, respectively. The area where the three ranges overlap simultaneously is located where both the steam temperature and running speed were low and when the spray depth is 9 cm and 12 cm (Figure 5 and Figure 6). In contrast, if the spray depth is 15 cm, the optimum range requires a high steam temperature and middle running speed level (Figure 7).

## 4. Discussion 

In this study, we investigated the removal efficiency of total nematodes and total bacteria in soil by high-temperature steam sterilization as well as the optimal operating conditions using the BBD. The BBD was used to design the experimental plans, and the empirical relationships between the three explanatory variables and responses were expressed by second-order polynomial equations using ANOVA. The explanatory variables were optimized using the desirability function. The optimized equation was achieved at a steam temperature of 150.56 °C, running speed of 1.69 m/min, and spray depth of 15.0 cm. These conditions predicted the responses as follows: nematode removal efficiency of 93.99%, bacteria removal efficiency of 97.49%, and oil consumption of 70.49 mL/m^2^.

Initially, soil disinfection methods were developed using chemicals, such as methyl bromide, or using steam sterilization [10]. For the chemical methods, pesticides, herbicides, and fumigants have been used to control weeds, plant diseases, and soilborne pathogens [23]. However, concerns about the widespread use of pesticides are increasing because of the effects of chemicals on the environment and human health [24,25,26]. Methyl bromide, one of the widely used soil fumigants, was phased out worldwide by 2015 because it damages the ozone layer [6,27]. Chloropicrin, which is widely used in greenhouses, has been demonstrated to be effective against several weeds, but it is also prohibited by the EU because it completely disturbs the bacterial community structure in soil and has a carcinogenic effect [28,29]. In addition, other chemical compounds, like dichloropropene and metam sodium, have also been banned or limited as a pesticide for agricultural purposes [10]. 

Because of the withdrawal of these chemical compounds, soil disinfection strategies using physical and biological methods are being developed. The new development methods produced in the current century are hot air treatment, radiation, and anaerobic soil disinfestation. Soil nematodes and some plant pathogens were not significantly removed by these new treatments in the early stages of development, but after continuous improvement, these methods resulted in higher crop yields than those obtained after steam sterilization [30,31,32,33,34,35,36]. For example, anaerobic soil disinfestation has been used on a commercial scale in California, USA, Japan, and some other countries [37]. In addition, steam sterilization, which has been used for more than a century, has the advantages of being relatively simple to use and effective against all nematodes, toxic pathogens, and weed seeds in the soil [9,10]. Recent advances in technology have made steam disinfectors smaller and capable of reliably dispensing high-temperature steam, making it easier for farming households to use steam sterilization at a low cost [12]. The steam sterilizer we used in our study costs about $ 0.13 to sterilize 1 m^2^ of soil. It is cheaper than the $ 0.17 using the most common fumigation agent in Korea. This approach may be an effective and economical soil disinfestation option for relatively small-scale farming systems across the world.

The most important priority during steam disinfection is to increase the temperature of the soil and maintain this temperature for a long time. Most insects, plant pathogens, viruses, and weed seeds in the soil will be destroyed if they are exposed to a temperature of 60 °C for 30 min [9]. Therefore, we should select the operating conditions that can maintain a sufficient temperature in the soil. In our study, it was the running speed of the steam disinfector that most significantly affected the temperature change of the soil. Figure 1 shows a significant decrease in the degree of increase in the soil temperature at a 15 cm depth, when the running speed was changed from 1 to 2 m/min. Similarly, the removal efficiencies of nematodes and total bacteria were significantly reduced when the running speed was increased. These results indicate that it is necessary to expose the soil to high-temperature steam for a sufficient time to effectively remove the total nematodes and total bacteria in soil. Another factor that affected the soil temperature increase was the spray depth. According to the results in Figure 1, steam sprayed at a 9 cm depth did not considerably change the temperature at 15 cm depth, because most of the steam rose to the top of the soil with the geothermal heat. Therefore, it is necessary to spray the steam deep into the soil, and for this purpose, the soil must be rotavated before conducting the steam disinfection, so that the nozzles spraying the high-temperature steam can be deeply embedded.

To our knowledge, this is the first study aiming to optimize the conditions of steam disinfection using the BBD. The BBD has been previously applied to optimize the removal efficiency of some heavy metals or residual chemical compounds in contaminated soil [38,39,40]. The findings of our study can be used as basic data for improving the disinfection efficiency and cost-effectiveness of high-temperature steam disinfection. However, the fact that our experiments were only performed in greenhouse soils with initial temperatures in the range of 12–14 °C is a limitation of this study. Further experimentation is required because the optimum conditions may change during summer or winter with higher or lower soil temperatures.

## 5. Conclusions

In this study, the Box–Behnken design was used to optimize and study the individual and interactive effects of the operation parameters of a high-temperature steam disinfector on oil consumption and removal efficiency of total nematodes and total bacteria in soil. The results of the ANOVA show that all response variables were fitted well by the quadratic model. The coefficients of determination ($R^{2}$), which indicate how much the model can predict variabilities in the response variables, were 0.9279, 0.9678, and 0.9979, respectively. The optimum conditions were found to be a steam temperature of 150.56 °C, running speed of 1.69 m/min, and spray depth of 15.0 cm, with a corresponding desirability value of 0.8367. These conditions predicted the responses as follows: nematode removal efficiency of 93.99%, bacteria removal efficiency of 97.49%, and oil consumption of 70.49 mL/m^2^.

## Figures and Tables

**Table 1:** Results of the level setting of the target parameters for the Box–Behnken design.

Variables	Symbols	Uncoded Levels		
		−1	0	1
Steam temperature (°C)	$x_{1}$	120	140	160
Running speed (m/min)	$x_{2}$	1.0	1.5	2.0
Spray depth (cm)	$x_{3}$	9	12	15

**Table 2:** Box–Behnken design with the experimental values and calculated results for the response variables in each experiment.

Run No.	Coded Levels of Explanatory Variables			Uncoded Levels of Explanatory Variables			Response Variables		
	$X_{1}$	$X_{2}$	$X_{3}$	$x_{1}$	$x_{2}$	$x_{3}$	Nematode RE ^†^ (%)	Total Bacteria RE ^†^ (%)	Oil Consumption (mL/m^2^)
1	0	−1	−1	140	1.0	9	94.42	96.56	145
2	0	1	−1	140	2.0	9	84.80	83.10	40
3	0	0	0	140	1.5	12	92.09	95.64	71
4	1	1	0	160	2.0	12	89.90	86.22	42
5	−1	0	1	120	1.5	15	91.77	94.30	58
6	1	0	−1	160	1.5	9	91.34	89.67	91
7	1	−1	0	160	1.0	12	97.25	94.95	191
8	0	−1	1	140	1.0	15	95.12	96.71	158
9	1	0	1	160	1.5	15	93.89	96.83	98
10	0	0	0	140	1.5	12	93.64	96.12	75
11	−1	−1	0	120	1.0	12	95.95	96.81	103
12	0	0	0	140	1.5	12	93.99	94.55	81
13	−1	1	0	120	2.0	12	82.56	80.23	31
14	0	1	1	140	2.0	15	92.01	94.58	49
15	−1	0	−1	120	1.5	9	90.29	88.55	41

^†^ RE: removal efficiency.

**Table 3:** Analysis of the models and regression coefficients for the three response variables.

Sources	Nematode Removal Efficiency				Total Bacteria Removal Efficiency				Oil Consumption			
	β	p-Value	$R^{2}$	$\mathrm{Adj}.\;R^{2}$	β	p-Value	$R^{2}$	$\mathrm{Adj}\;R^{2}$	β	p-Value	$R^{2}$	$\mathrm{Adj}.\;R^{2}$
Model		0.022	0.9279	0.7982		0.003	0.9678	0.9097		<0.001	0.9979	0.9941
Lack-of-fit		0.189				0.142				0.876		
Constants	90.0	<0.001			−1.2	<0.001			−469	<0.001		
$x_{1}$	0.350	0.568			1.883	0.088			8.04	<0.001		
$x_{2}$	−30.2	0.007			−27.5	0.002			−83.8	<0.001		
$x_{3}$	−0.09	0.087			−2.75	0.006			5.31	0.037		
$x_{1}^{2}$	−0.00199	0.428			−0.00786	0.014			−0.01240	0.049		
$x_{2}^{2}$	−4.12	0.315			−10.97	0.023			84.17	<0.001		
$x_{3}^{2}$	−0.069	0.529			0.0048	0.962			0.144	0.529		
$x_{1}\cdot x_{2}$	0.1510	0.149			0.1962	0.061			−1.925	<0.001		
$x_{1}\cdot x_{3}$	0.0045	0.775			0.0059	0.684			−0.0417	0.231		
$x_{2}\cdot x_{3}$	1.085	0.126			1.888	0.018			−0.67	0.609		

**Table 4:** Optimal operation parameters for the three explanatory variables.

Variables	Optimal Conditions			Predicted Responses		
	Steam Temperature (°C)	Running Speed (m/min)	Spray Depth (cm)	Nematode RE ^†^ (%)	Bacteria RE ^†^ (%)	Oil Consumption (mL/m^2^)
Values	150.56	1.69	15.0	93.99	97.49	70.49

^†^ RE: removal efficiency.

## Supplementary Materials

The following are available online at https://www.mdpi.com/1660-4601/17/14/5029/s1, Figure S1: Flow chart of the optimization design.
