# Supplementary figures and images for: Optimizing Operating Parameters of High-Temperature Steam for Disinfecting Total Nematodes and Bacteria in Soil: Application of the Box–Behnken Design
Source: Int J Environ Res Public Health. 2020 Jul 13;17(14):5029. doi: 10.3390/ijerph17145029 (PMC7400156; doi:10.3390/ijerph17145029)

## Supplementary Material

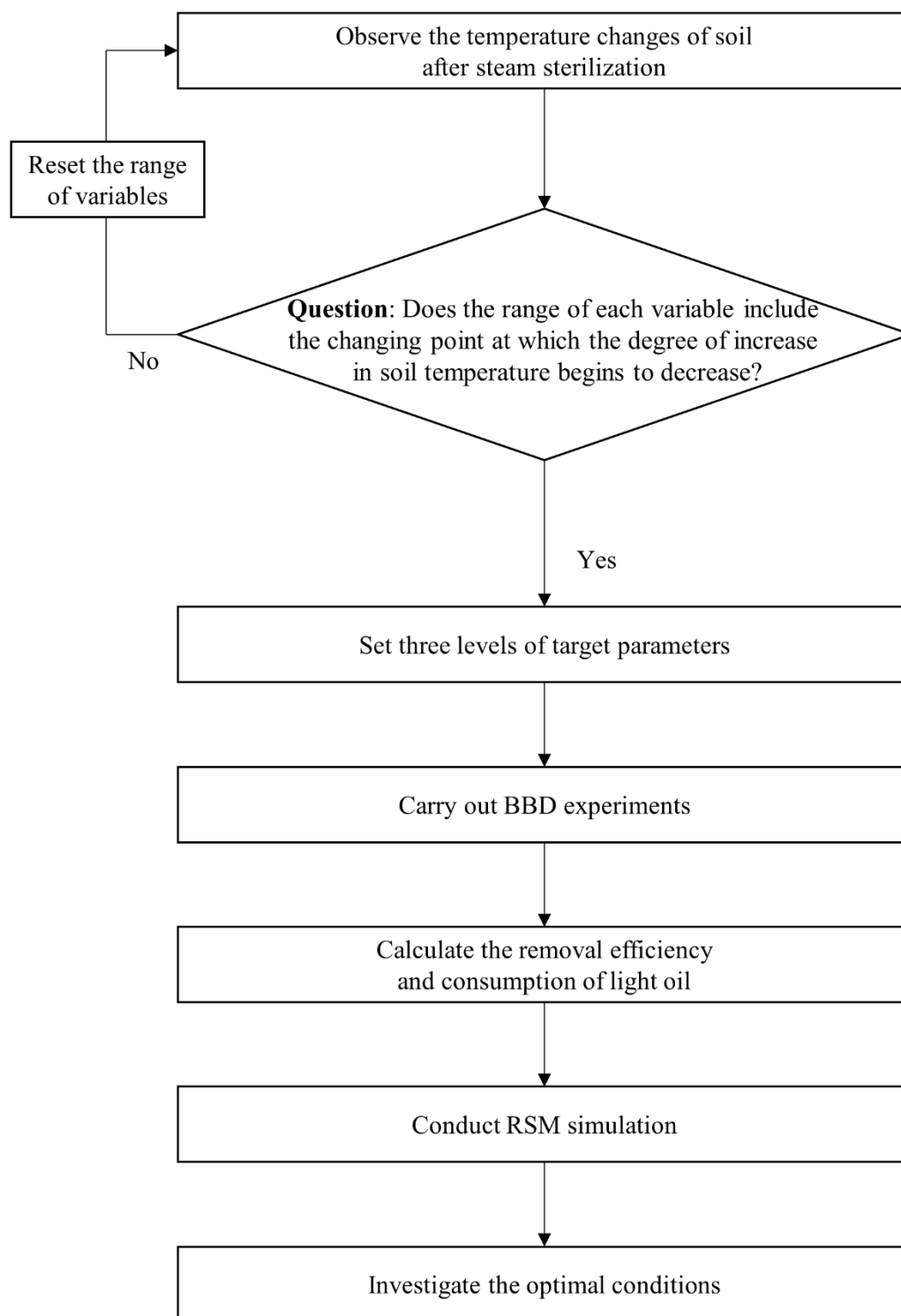

**Figure S1.** Flow chart of the optimization design.

Supplement: Supplementary file 1 [file ijerph-17-05029-s001.pdf]
